# Personal dose equivalent *H*_p_(0.07) during ^68^Ga-DOTA-TATE production procedures

**DOI:** 10.1007/s00411-022-01015-y

**Published:** 2023-01-10

**Authors:** M. Wrzesień, Ł. Albiniak

**Affiliations:** Department of Nuclear Physics and Radiation Safety, Faculty of Physics and Applied Informatics, University of Lodz, Pomorska 149/153, 90-236 Lodz, Poland

**Keywords:** Nuclear medicine, Dosimetry, *H*_p_(0.07), Hand exposure, Ga-68, Radiation protection, 68 Ga-DOTA-TATE

## Abstract

This work presents the exposure of hands of the personnel of a nuclear medicine department who prepare and administer ^68^Ga-DOTA-TATE. Dosimetry measurements were performed during three 1-week sessions, for nine production procedures. A total of 360 measurements were made by using high-sensitivity MCP-N thermoluminescent detectors. Annealed detectors were and vacuum-packed in foil and then placed on each fingertip of both hands of five radiochemists and four nurses (one detector for one fingertip). The greatest exposure to ionizing radiation was found on the non-dominant left hand of radiochemists and nurses. A maximum *H*_p_(0.07)/A value of 49.36 ± 4.95 mSv/GBq was registered for radiochemists during the ^68^ Ga-DOTA-DATE activity dispensing procedure. For nurses performing the radiopharmaceutical injection procedure, a corresponding maximum value of 1.28 ± 0.13 mSv/GBq was measured, while the mean value for all the nurses was 0.38 mSv/GBq. The dispensing procedure accounted for approximately 60% of the total exposure of radiochemists' fingertips. Based on the results obtained it is recommended that a ring dosimeter should be routinely placed on the middle finger of the non-dominant hand of radiochemists and nurses. Furthermore, it is proposed to systematically train workers in handling open sources of ionizing radiation, with the aim of reducing the required handling time.

## Introduction

It has been known for a long time that in the case of nuclear medicine procedures, the hands of personnel are the most exposed parts of the body (Kollaard et al. 2021). This is because employees typically do not only carry out the procedures of producing the radionuclides by themselves, but also label chemical compounds with these radionuclides in order to obtain radiopharmaceuticals, and/or inject the prepared radiopharmaceutical into a patient’s body (Stuarto 1990; Chiesa et al. 1997; Williams et al. 1990; Batchelor et al. 1991; Dhanse et al. 2000; Kubo & Mauricio 2014; Sandouqa et al. 2011; Leide-Svegborn 2011). The occupational groups most affected by this exposure are radiochemists and nurses (Meisenheimer et al. 2020; Wrzesień and Napolska 2015; Wrzesień et al. 2019; Wrzesień and Albiniak 2016; Wrzesień 2018; Vanhavere et al. 2012). During the last decade, a lot of attention in scientific publications has been devoted to the exposure of the hands when performing tasks involving radionuclides (Sans-Merce et al. 2011a; Breeman et al. 2005; Wrzesień et al. 2008; Carnicer et al. 2011; Sans Merce et al. 2011b). However, it is important to emphasize that nuclear medicine involves a multitude of radionuclides and procedures that are carried out, for example, as part of the production and quality control of radiopharmaceuticals. Consequently, the method of producing the radionuclide of interest should also be taken into account. This includes, for example, the production of radiopharmaceuticals using nuclear reactors, cyclotrons and short-lived nuclide generators.

Short-lived nuclide generators, in particular the ^99^Mo/^99m^Tc generator which is the main source of ^99m^Tc, one of the diagnostic isotopes most commonly used in nuclear medicine, allow for a rather uncomplicated production of the radionuclide of interest. Compared to the ^99^Mo/^99m^Tc generator, the operation of the ^68^Ge/^68^ Ga short-lived nuclide generator is more complicated (Chiesa et al. 1997). Therefore, the question remains whether the production of ^68^ Ga with a ^68^Ge/^68^ Ga generator would result in a greater radiological exposure for personnel performing elution and labelling of chemical compounds involving ^68^ Ga.

Nowadays the ^68^Ge/^68^ Ga generator and DOTA-conjugated peptides are not registered for trading on the market. Therefore, these peptides must be prepared taking into account national regulations as well as the Good Radiopharmaceutical Practice (GRPP) described in the detailed guidelines of the European Society for Nuclear Medicine (EANM) (Piciu 2012; cGRPP; Elsinga et al. 2010; ISO 1999).

Recently, exposure of the eye lenses of nuclear medicine staff due to the preparation and injection of DOTA-TATE radiopharmaceuticals labelled with ^68^ Ga was discussed (Wrzesień and Albiniak 2018). In that publication it was underlined that manual operation of the ^68^Ge/^68^ Ga generator and of ^68^ Ga sources as well as labelling procedures and activity separation may be a source of exposure of the eye lenses, which may lead to exposures higher than the annual dose limit for the eye lens. Consequently, in the present study it was investigated whether similar conclusions may be reached with regard to the exposure of the hands of staff performing procedures involving ^68^ Ga and the ^68^ Ga-DOTA-TATE radiopharmaceutical.

## Material and methods

Dosimetric measurements were performed with high-sensitivity thermoluminescent detectors (TLDs) (LiF: Mg, Cu, P) (produced by RADCARD in Poland) in one out of three national nuclear medicine centres, where procedures for the diagnosis of neuroendocrine tumours using the PET technique are performed. Five radiochemists and four nurses participated in 3-week measurement sessions (dosimetric data were obtained for nine full production procedures). Before starting the measurements, the detectors were annealed according to the manufacturer's recommendations, and each detector was individually vacuum-packed in foil. The detectors prepared in this way were placed on the workers' fingertips on the both of the hands. The necessary condition for the implementation of dosimetric measurements was the lack of any influence of the presence of detectors placed on the fingertips on the time and efficiency of the procedures performed by radiochemists and nurses. In total, 360 measurements were made with TLD detectors.

A gamma radiation source including ^137^Cs (^60^Co/^137^Cs irradiator) was used to calibrate the detectors in the Secondary Standard Dosimetry Laboratory located at the Prof. J. Nofer Institute of Occupational Medicine in Lodz, Poland. Dosemeters were calibrated in accordance with ISO 4037-3 (ISO 1999) in the range from 0.05 to 30 mGy air kerma. The *H*_p_(0.07) for fingers was calculated taking into account the conversion coefficient *h*_pK_(0.07) given in the ISO International Standard (ISO 1999). An ISO acrylic rod phantom compliant with ISO 4037 Part 3:1999 was used as well. In the dosimetric measurements, background radiation was taken into account. The thermoluminescent detectors were read using the RA '04 reader, manufactured by the Polish company Mikrolab (http://www.tld.com.pl/). The determined measurement uncertainty of the dose was 5%, respectively, while for the determination of the activity value the error was 10%.

### Procedures for production and injection of ^68^ Ga-DOTA-TATE

The ^68^ Ga-DOTA-TATE procedure typically begins with the elution of the ^68^Ge/^68^ Ga generator. The speed of elution should be no more than 2 ml/min. The eluate ends its path in a vial containing the peptide. The elution procedure is completed by measuring the activity of the eluate obtained from the ^68^Ge/^68^ Ga generator. The second stage—labelling—is preceded by heating the preparation at 100 °C for 10 min. After heating, the peptide is passed through an activated C-18 cartridge to separate the water fraction. Column-tied preparation ^68^ Ga-DOTA-TATE using ethanol is then washed into a vial, thus forming the so-called ethanol phase. Both procedures are completed by measuring the activity in the water and the ethanol phase. Then the activity administered to the patient is measured. A portion of activity of the labelled radiopharmaceutical is taken through a sterile filter. After injecting the radiopharmaceutical to the patient, the syringe used is returned to the calibrator to measure the activity remaining in the syringe.

### Statistical analysis

In the study *H*_p_(0.07)/A was measured (i.e., the normalized personal dose equivalent). The values of activity (A) used were the actual values for each procedure that was carried out. Statistical analysis was performed with the Mann–Whitney test using STATISTICA v. 10.0.MR1. Any differences found were considered statistically significant if the p-value was below 0.05. Minimum, mean and maximum values are also presented for all the procedures, as well as 25^th^ and 75^th^ percentiles.

## Results

**Fig. 1:**
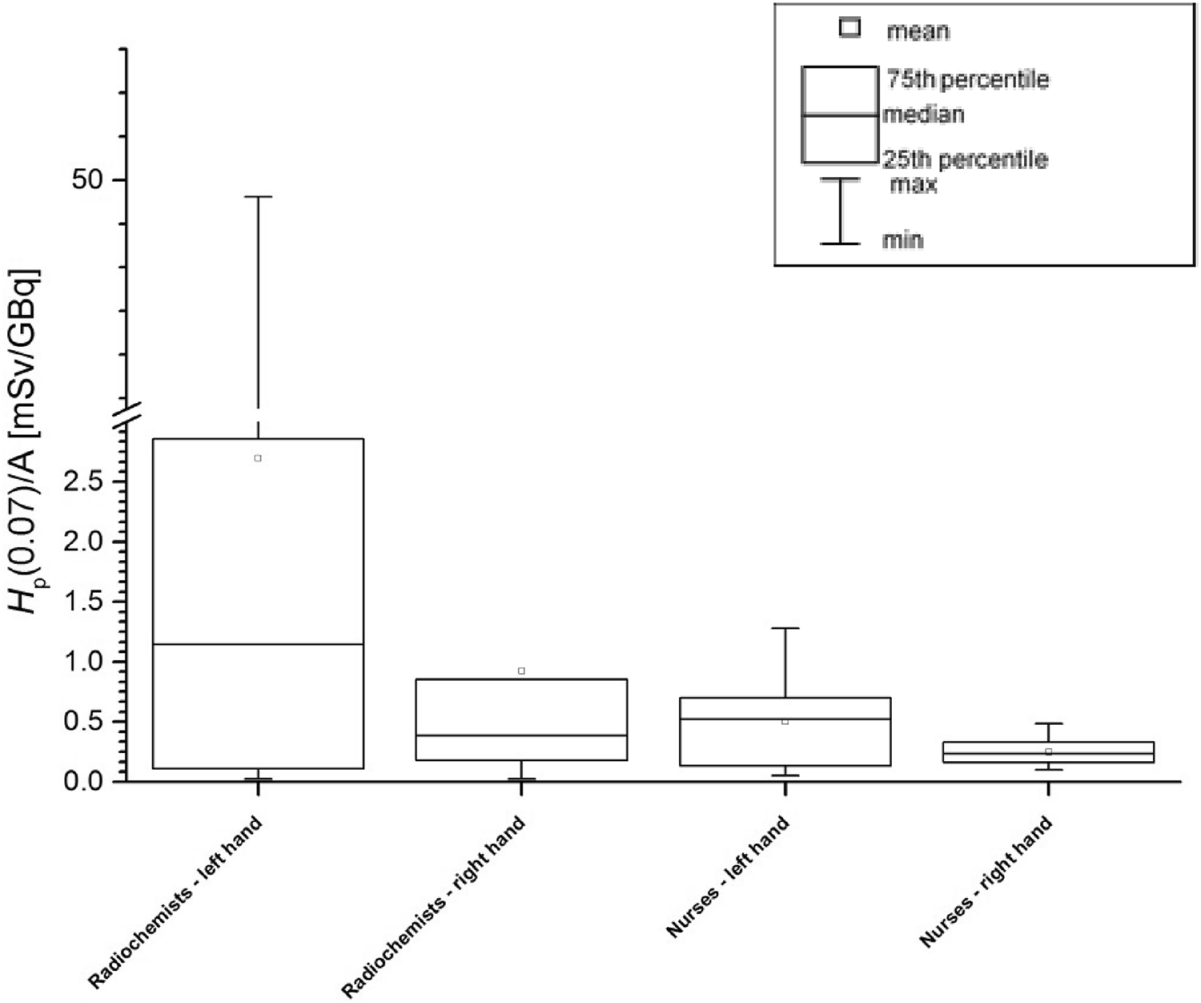
Exposure of the right and left hand in terms of *H*_p_(0.07) values per unit activity (A) of radiochemists responsible for the ^68^ Ga-DOTA-TATE production process and of nurses performing injection of radiopharmaceuticals to the patients

Figure 1 presents *H*_p_(0.07)/A values measured with the TLDs for left and right hands of radiochemists and nurses.

The maximum *H*_p_(0.07)/A value of 49.36 ± 4.95 mSv/GBq was recorded for the index finger of the left hand of Radiochemist 1 during the procedure of dispensing a dose of the radiopharmaceutical. For nurses the maximum *H*_p_(0.07)/A value of 1.28 ± 0.13 mSv/GBq was recorded for the thumb of the left hand of Nurse 1. This suggests that the dosimetric ring should be worn by staff on the left hand (on the non-dominant hand), because for both groups of workers higher doses were recorded for the left than for the right hand.

**Fig. 2:**
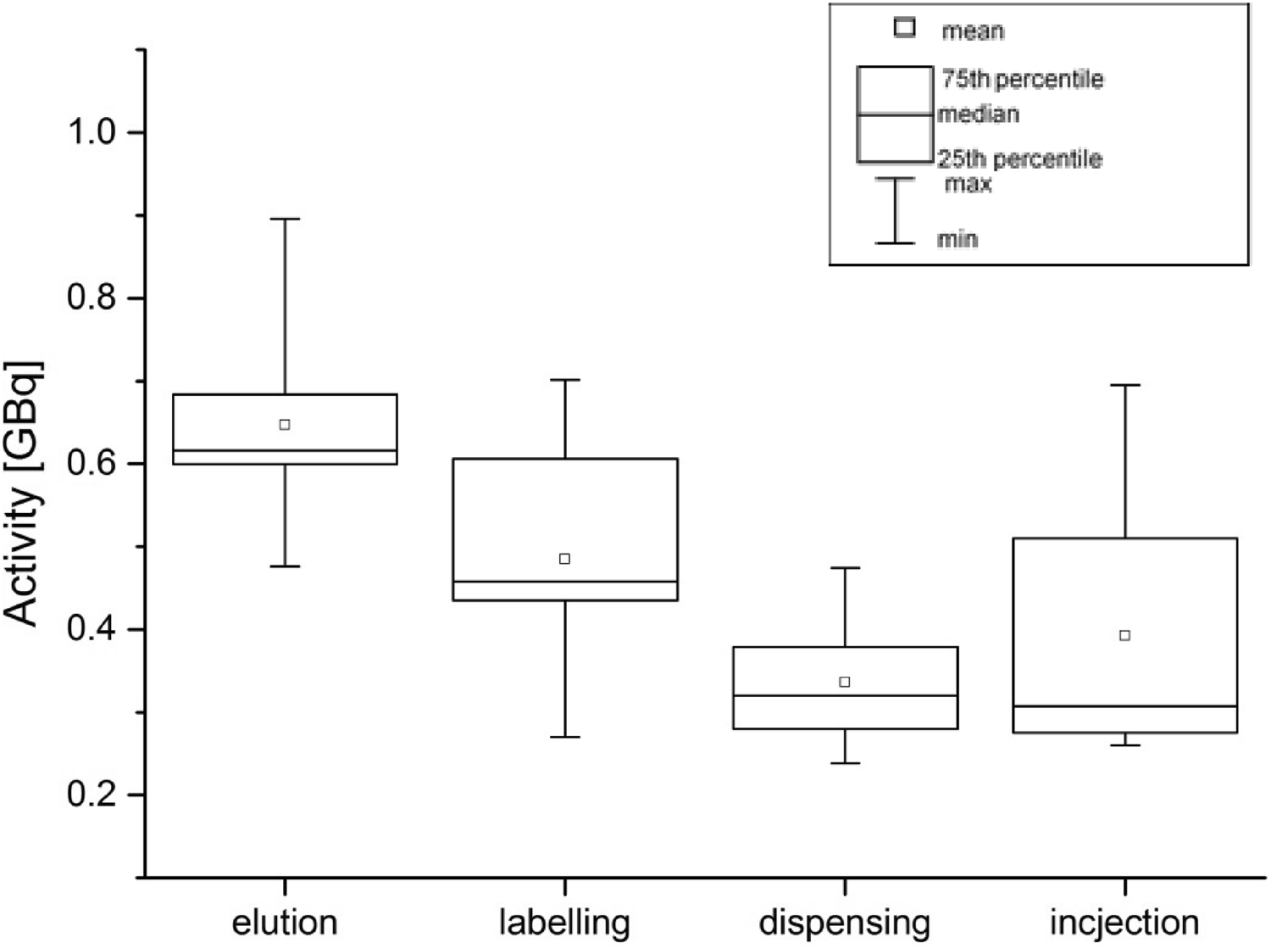
Activities of ^68^ Ga-DOTA-TATE at particular stages of production and during injection

Figure 2 shows the activity used for the individual production stages of ^68^ Ga-DOTA-TATE and during the injection of the radiopharmaceutical.

The highest activity of the radiopharmaceutical with which the radiochemists worked was recorded for the elution stage—0.90 GBq, while the lowest activity was recorded for nurses during the injection of the radiopharmaceutical—0.26 GBq.

### Fingertip exposure during ^68^Ge/^68^ Ga generator elution

Table 1 presents statistical processing of the exposure data of radiochemists' fingertips during the radiopharmaceutical elution procedures.

**Table 1:** *H*_p_(0.07)/*A* values measured on the fingertips of radiochemists during the elution procedure

		*H*_p_(0.07)/*A* [mSv/GBq]				
		Minimum value	Maximum value	Mean value	25th percentile	75th percentile
Left hand	Thumb	0.02	2.39	0.32	0.05	0.09
	Index finger	0.02	4.09	0.50	0.03	0.08
	Middle finger	0.02	0.83	0.16	0.03	0.11
	Ring finger	0.02	0.37	0.10	0.03	0.09
	Small finger	0.02	0.12	0.05	0.03	0.07
Right hand	Thumb	0.07	0.38	0.19	0.14	0.26
	Index finger	0.02	0.35	0.15	0.08	0.22
	Middle finger	0.02	0.29	0.16	0.11	0.19
	Ring finger	0.02	0.22	0.11	0.05	0.14
	Small finger	0.03	0.20	0.08	0.04	0.09

The obtained doses confirm that the left hand of workers is exposed to higher doses of radiation than the right hand. This is especially true of the thumb and index finger. In the case of the right hand fingertips, the spread of the average values obtained is not so large as for the left hand fingertips.

**Fig. 3:**
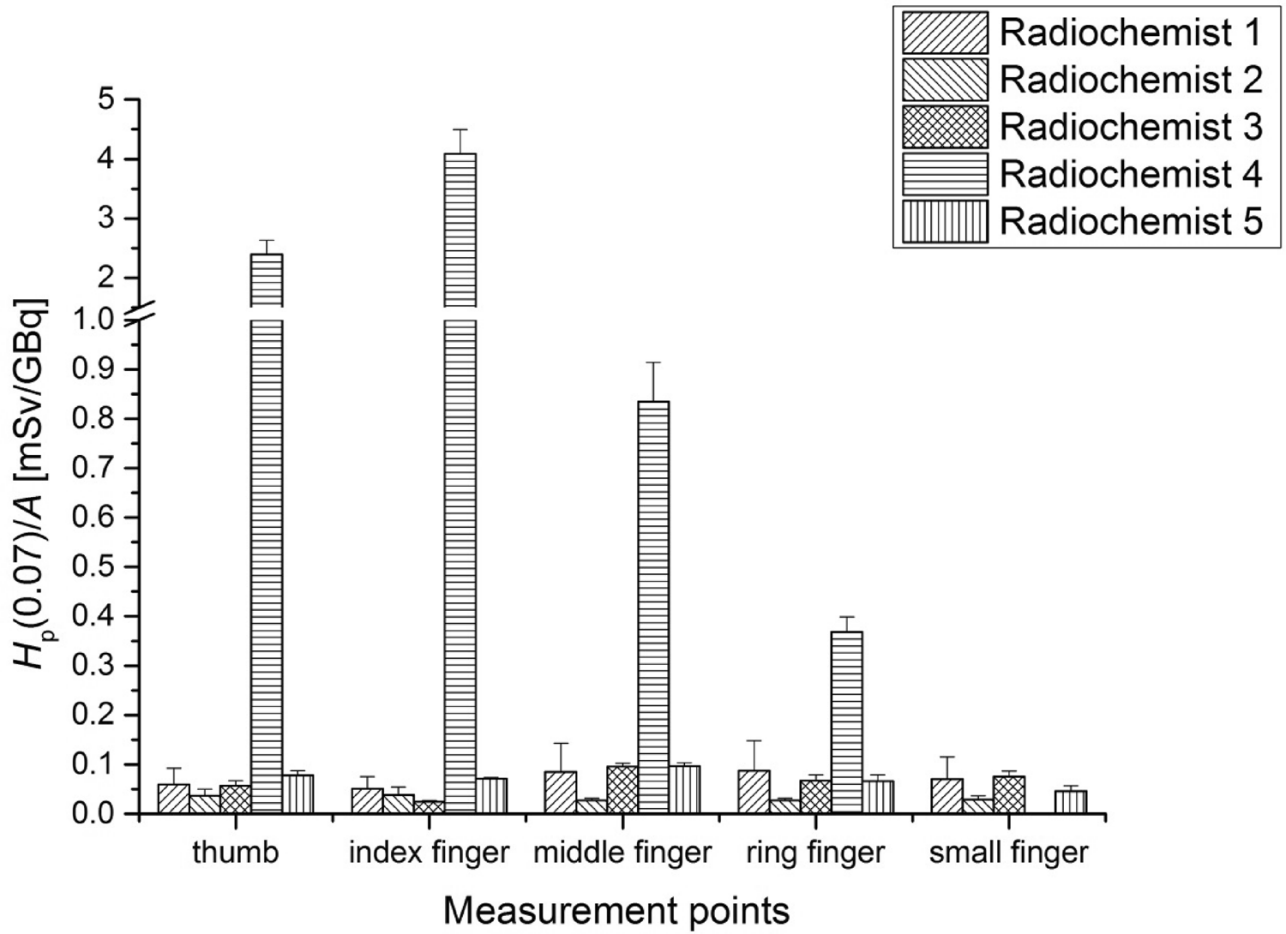
Average *H*_p_(0.07) values per unit activity (A) of ^68^ Ga-DOTA-TATE (with standard errors) measured at fingertips of the left hand for radiochemists performing the elution of the ^68^Ge/^68^ Ga generator

Figure 3 shows fingertip doses of the left hand of the radiochemists during the ^68^Ge/^68^ Ga generator elution. Dose values for the fingertips of the right hands of the radiochemists are not shown because the highest value recorded for the right hand (for the thumb of Radiochemist 1—0.38 ± 0.11 mSv/GBq) is 11 times smaller than the highest value recorded for the fingers of the left hand (index finger of Radiochemist 4—4.09 ± 0.02 mSv/GBq).

The highest value of *H*_p_(0.07)/A was recorded for the index finger of the left hand of Radiochemist 4—4.09 ± 0.02 mSv / GBq. The average value of the activity with which Radiochemist 4 worked was 0.65 GBq.

### Labelling the pharmaceutical with ^68^ Ga

For the labelling procedure, the highest doses per unit activity were recorded for the fingertips of Radiochemist 2 and Radiochemist 5. For the rest of the staff *H*_p_(0.07)/A values did not exceed a value of 1.16 ± 0.55 mSv/GBq for the fingertips of the left hand and 0.55 ± 0.10 mSv/GBq for the fingertips of the right hand. Table 2 presents minimum, maximum, mean, 25th percentile and 75th percentile values for both hands of all radiochemists.

**Table 2:** *H*_p_(0.07)/*A* values measured on the fingertips of radiochemists during the labelling procedure

		*H*_p_(0.07)/*A* [mSv/GBq]				
		Minimum value	Maximum value	Mean value	25th percentile	75th percentile
Left hand	Thumb	0.85	6.56	2.10	1.16	1.51
	Index finger	0.51	6.70	2.13	0.85	1.83
	Middle finger	0.38	11.56	2.29	0.64	1.34
	Ring finger	0.39	1.56	0.91	0.69	1.00
	Small finger	0.45	5.65	1.89	0.89	1.49
Right hand	Thumb	0.28	5.81	1.54	0.33	0.67
	Index finger	0.20	1.92	0.62	0.27	0.49
	Middle finger	0.06	3.13	0.74	0.22	0.84
	Ring finger	0.16	1.89	0.52	0.17	0.54
	Small finger	0.17	1.11	0.45	0.26	0.51

The lowest Hp(0.07)/A value was recorded for the middle finger of the right hand and was 0.06 mSv/GBq. For the left hand, it was the TLD of the middle finger that recorded the lowest value—0.38 mSv/GBq (i.e., more than 6.3 times that of the same finger of the right hand).

**Fig. 4:**
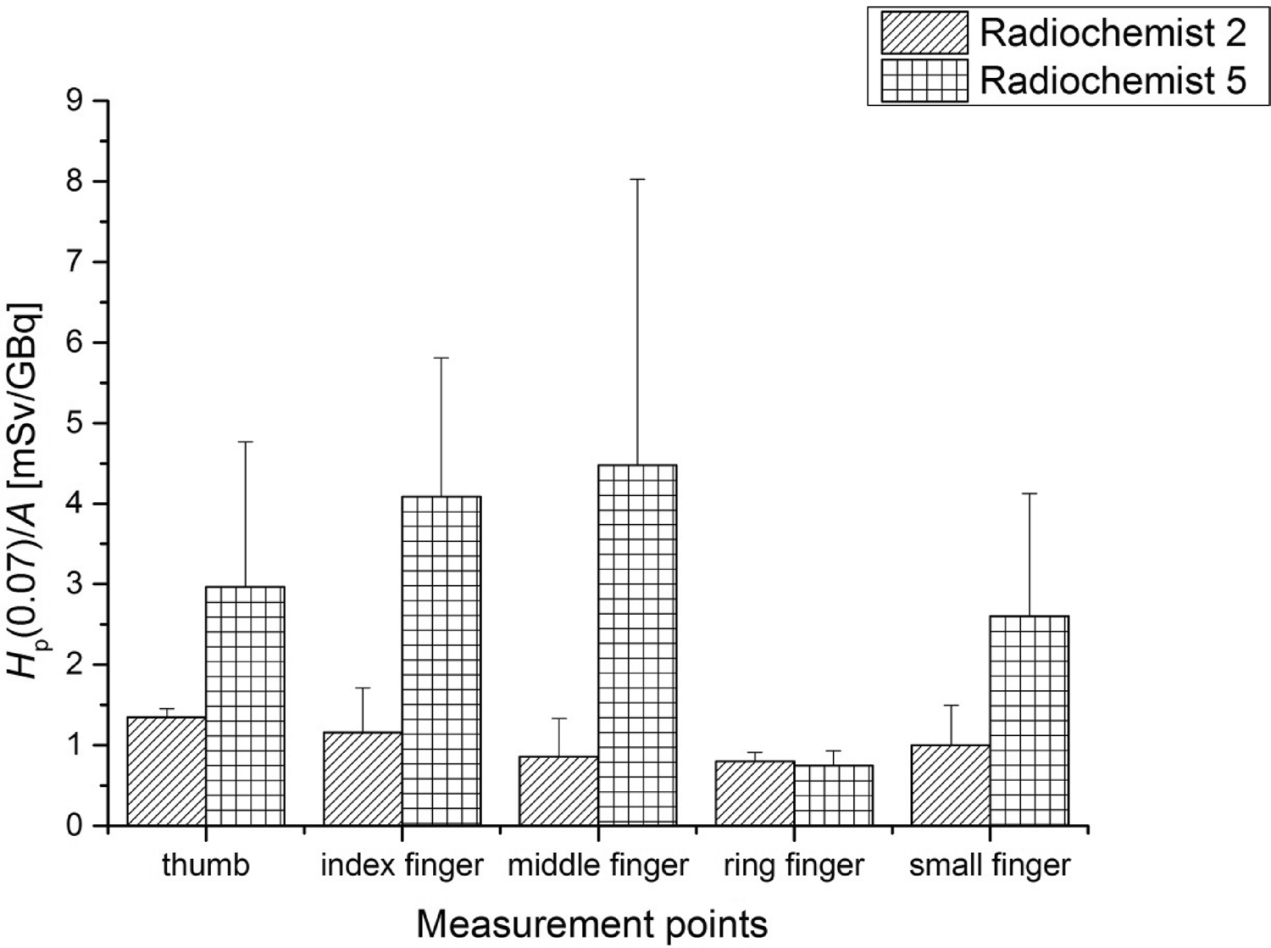
Average values of *H*_p_(0.07) per unit activity (A) of ^68^Ga-DOTA-TATE (with standard errors) measured at the left hand of employees while labelling the pharmaceutical with the ^68^Ga

In Fig. 4 the results obtained for the two most exposed workers are shown.

The highest *H*_p_(0.07) dose per unit activity was recorded for the middle finger of the left hand of Radiochemist 5 (11.56 ± 3.54 mSv/GBq). In contrast, for Radiochemist 2 the highest *H*_p_(0.07) dose per unit activity was measured for the small finger of the left hand (4.54 ± 1.61 mSv/GBq). During the labelling procedure, the average activity was 0.46GBq.

### Dispensing the radiopharmaceutical

Table 3 shows data obtained during the dispensing procedures.

**Table 3:** *H*_p_(0.07)/*A* values measured at the fingertips of radiochemists during the dispensing procedure

		Hp(0.07)/A [mSv/GBq]				
		Minimum value	Maximum value	Mean value	25th percentile	75th percentile
Left hand	Thumb	0.76	22.73	7.68	1.08	21.02
	Index finger	0.09	49.36	13.88	0.60	28.56
	Middle finger	0.59	7.46	2.77	0.99	3.98
	Ring finger	0.60	4.82	2.37	0.65	4.31
	Small finger	0.06	4.22	2.00	0.36	2.81
Right hand	Thumb	0.08	9.38	2.96	0.84	7.32
	Index finger	0.64	6.99	2.38	1.36	2.42
	Middle finger	0.06	9.29	2.77	0.41	6.31
	Ring finger	0.07	5.90	1.93	0.56	4.70
	Small finger	0.06	0.87	0.46	0.35	0.54

As in previous cases, it was for the fingertips of the left hand that showed higher values.

**Fig. 5:**
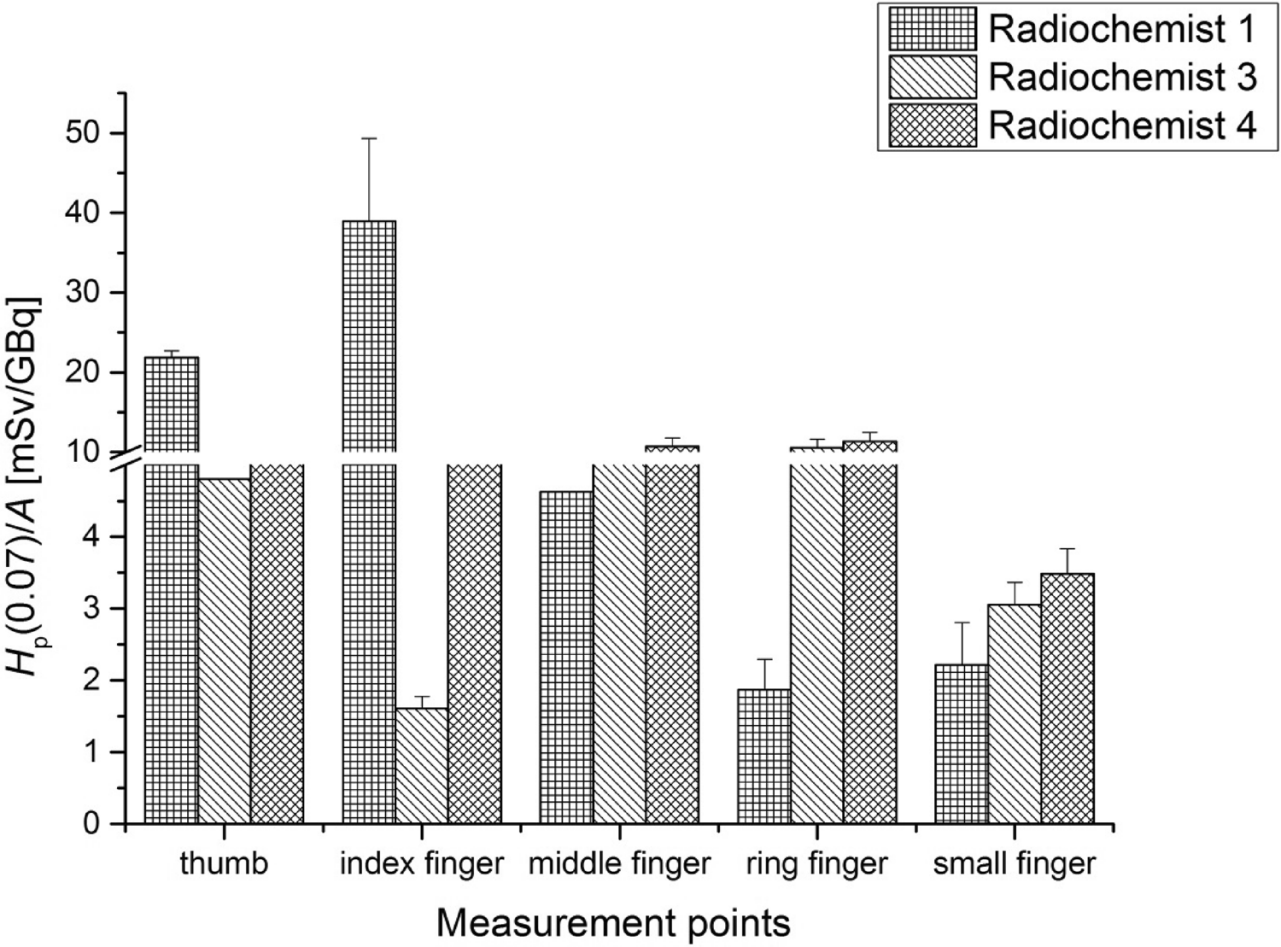
Average values of *H*_p_(0.07) per unit activity (A) (with standard errors) recorded at all fingers of the left hand for radiochemists dispensing ^68^Ga-DOTA-TATE for the patients

Exposure characteristics of the thumb and index finger were also noted for the dispensing procedure. Figure 5 presents the corresponding averaged *H*_p_(0.07)/*A* values for the three radiochemists**.**

In the case of the procedure of dispensing the activity of the radiopharmaceutical, the largest *H*_p_(0.07)/A value (38.96 ± 10.40 mSv/GBq) was noted for the index finger of the left hand of Radiochemist 1. The average activity during the procedure of dispensing the dose of ^68^ Ga-DOTA-TATE was 0.31 GBq.

### Injection of ^68^Ga-DOTA-TATE

In the case of nurses, higher *H*_p_(0.07) values per unit activity were registered for the left hand fingertips than for right hand fingertips. The lowest dose was measured at the small finger of the left hand (0.05 mSv/GBq), while the highest doses were measured for the thumb and index finger of the left hand (1.28 and 1.26 mSv/GBq, respectively) (Table 4).

**Table 4:** *H*_p_(0.07)/*A* values measured at the fingertips of nurses during the injection procedure

		*H*_p_(0.07)/*A* [mSv/GBq]				
		Minimum value	Maximum value	Mean value	25th percentile	75th percentile
Left hand	Thumb	0.54	1.28	0.80	0.66	0.84
	Index finger	0.54	1.26	0.85	0.59	1.06
	Middle finger	0.10	1.20	0.45	0.15	0.54
	Ring finger	0.06	0.50	0.20	0.07	0.27
	Small finger	0.05	0.55	0.21	0.06	0.28
Right hand	Thumb	0.22	0.49	0.31	0.24	0.35
	Index finger	0.14	0.34	0.21	0.17	0.22
	Middle finger	0.20	0.39	0.29	0.22	0.37
	Ring finger	0.13	0.39	0.24	0.13	0.34
	Small finger	0.10	0.29	0.19	0.10	0.26

**Fig. 6:**
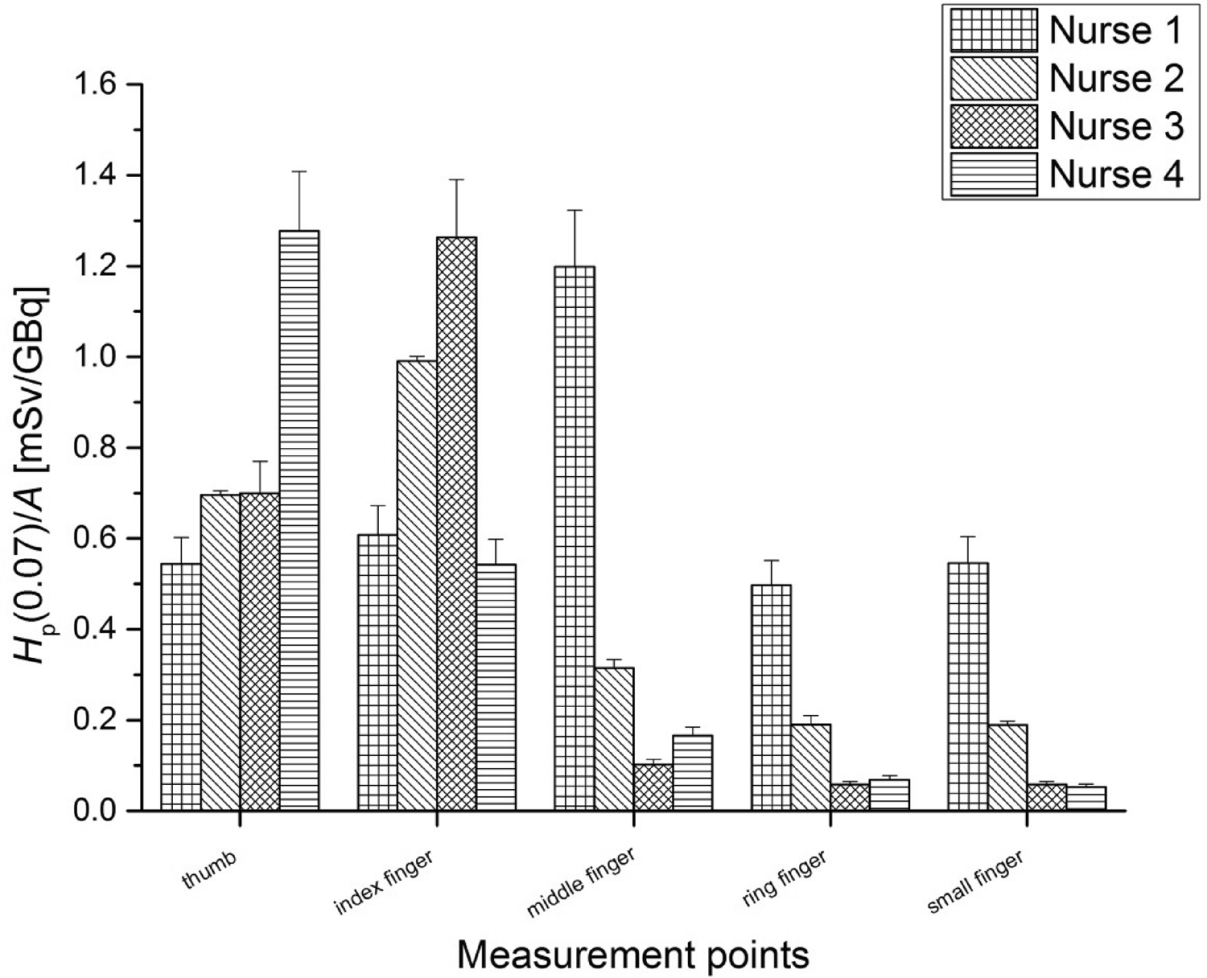
Average values of *H*_p_(0.07) per unit activity (A) (with standard errors) measured at fingertips of the left hand for nurses during the injection of ^68^Ga-DOTA-TATE for the patients

**Fig. 7:**
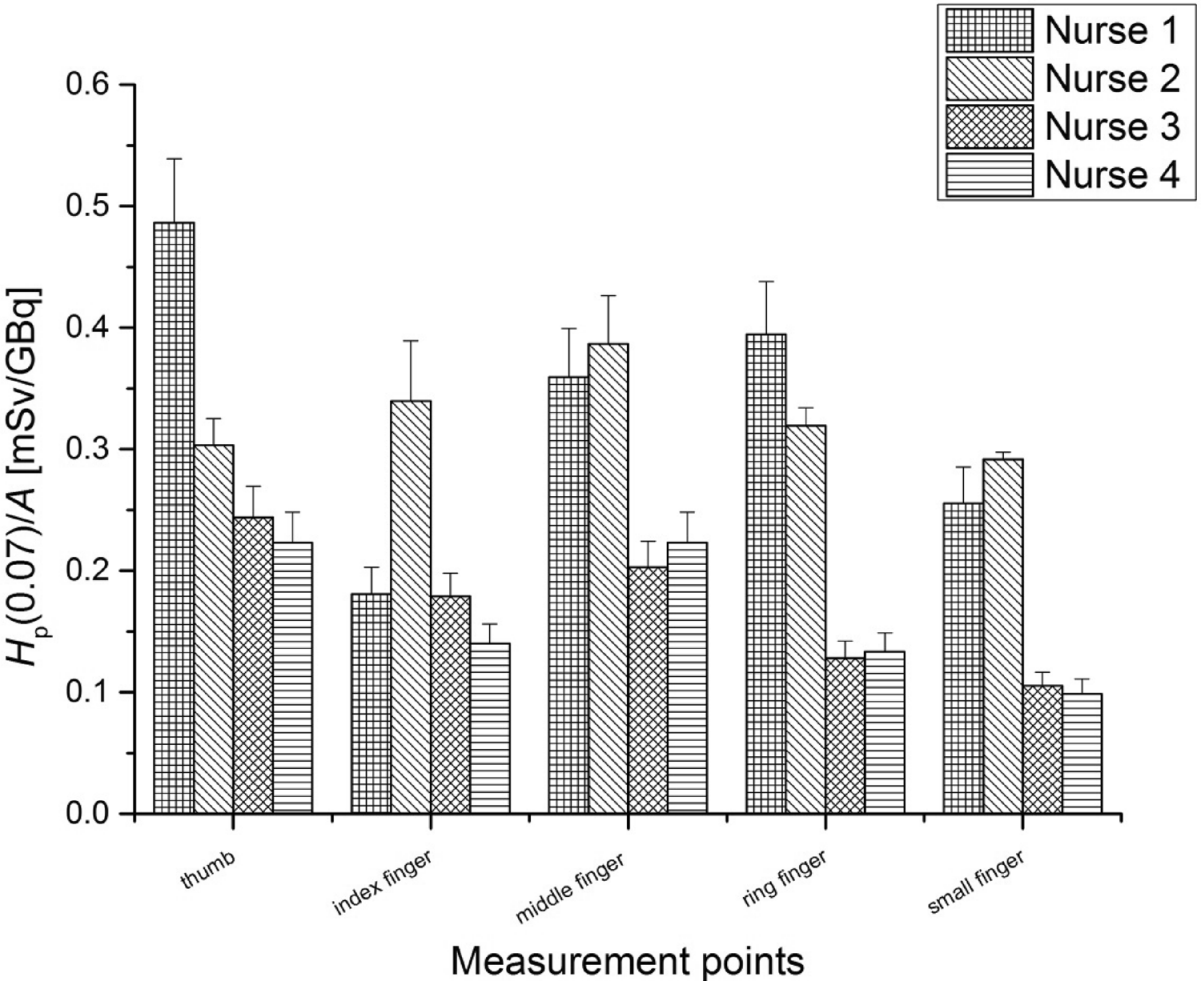
Average values of H_p_(0.07) per unit activity (A) (with standard errors) measured at fingertips of the right hand for nurses during the injection of ^68^Ga-DOTA-TATE for the patients

Figures 6 and 7 present the average values of *H*_p_(0.07)/*A* measured for nurses performing the radiopharmaceutical injection procedure, for the left and right hand, respectively.

The highest *H*_p_(0.07) value per unit activity was registered for the thumb of the left hand of Nurse 4 at 1.28 ± 0.13 mSv/GBq. The mean value of the activity injected to the patient was 0.39GBq. In this case, measurements showed that the highest mean dose of the right hand fingertips were about half the mean dose of the left hand fingertips.

## Discussion

In nuclear medicine a wide range of radionuclides are used, which emit different types of radiation with different radiation energy, and which show different physical half-lives. At first glance it might appear that the situation is similar for ^18^F, ^68^Ga, and ^99m^Tc. It is worth noting, however, that procedures with ^99m^Tc are performed manually, similar to the procedures with ^68^Ga. Moreover, both ^99m^Tc and ^68^Ga are generator products—for ^99m^Tc it is a ^99^Mo/ ^99m^Tc generator, while for ^68^ Ga it is a ^68^Ge/ ^68^ Ga generator. Of course, in both cases the energy of the gamma radiation emitted by ^99m^Tc and ^68^ Ga is different. Therefore, it seems that it is rather difficult to find a common "denominator" for both radionuclides compared to the ^18^F. It is noted that ^18^F is produced by means of cyclotrons, and moreover, the process of labelling the carrier with ^18^F is carried out automatically. Nevertheless, also for ^18^F quality control procedures of the produced radiopharmaceutical are performed manually. Moreover, the energy of the gamma radiation resulting from the annihilation process of positrons produced by the decay of ^18^F and ^68^ Ga 511 keV.

^68^ Ga is a radioisotope which is the basis of, among others, the radiopharmaceutical compound ^68^ Ga-DOTA-TATE. This radioisotope generates positrons during decay which annihilate with electrons to produce gamma radiation with an energy of 511 keV. These photons are the basis of positron emission tomography. In fact, they are the main source of exposure to ionizing radiation for the hands of medical personnel working in the production and injection of ^68^ Ga-DOTA-TATE.

**Fig. 8:**
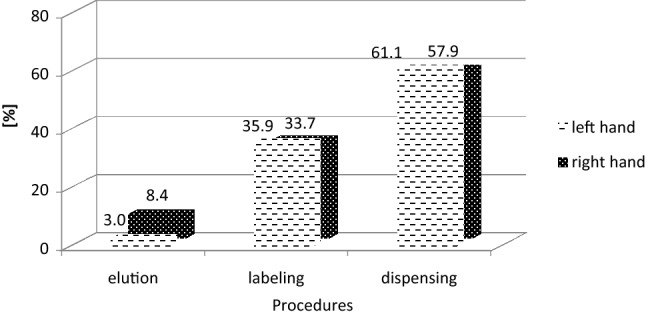
Percentage of total *H*_p_(0.07) dose per unit activity obtained during the various steps of ^68^ Ga-DOTA-TATE preparation

First process in its production is elution, where ^68^ Ga is eluted from the column of the generator. The next step is the labelling process, where the eluate with the ^68^ Ga is combined with a chemical substance. Last step before injecting the radiopharmaceutical to the patient is the procedure of dispensing the activity of ^68^ Ga-DOTA-TATE. Figure 8 shows the percentage of the total dose for each production step of ^68^ Ga-DOTA-TATE for the left and right hand of radiochemists.

The obtained results show that in the case of ^68^ Ga-DOTA-TATE production, the procedure of dispensing has the biggest impact on the exposure of the workers’ fingertips. On the other hand, the lowest percentage of total *H*_p_(0.07)/A was obtained in the elution process. It is worth mentioning that according to Fig. 2, the highest ^68^ Ga activity values with which radiochemists work are obtained during elution of the generator, while the lowest activity occurs during dispensing. However, taking into account that the dispensing procedure is the most time-consuming process, and that the elution process is the fastest procedure in the whole process, means that the duration of the procedure (in accordance with the basic principles of radiation protection) is the factor that dominates the radiation doses received by the workers.

**Fig. 9:**
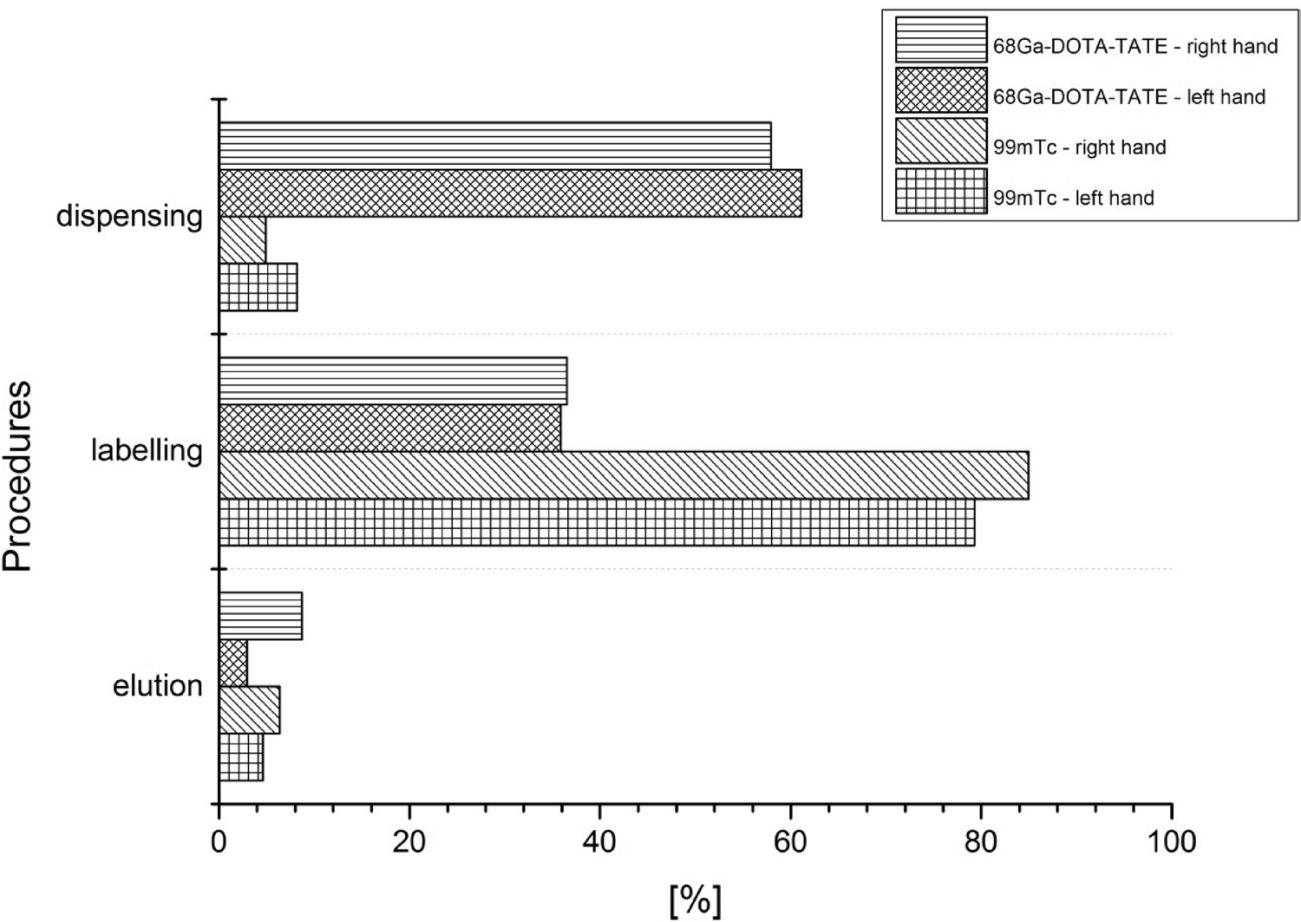
Percentage of total *H*_p_(0.07) dose per unit activity (A) for the left and right hand of radiochemists involved in the production of ^68^ Ga-DOTA-TATE and ^99m^Tc (results for ^99m^Tc were taken from Wrzesień 2018)

Previously, the authors of the present study have already investigated hand exposures due to the most popular radionuclide, ^99m^Tc, produced with a radionuclide generator (Wrzesień 2018). It is worth noting that compared with ^18^F or ^68^ Ga, ^99m^Tc is a source of gamma radiation with an energy of 144 keV. However, the similar way in which radiopharmaceuticals—^68^ Ga-DOTA-TATE and ^99m^Tc—are produced, based on short-lived radionuclide generators, could indicate that the greatest exposure in both cases will be in the labelling process (as is the case with ^99m^Tc). Figure 9 compares the percentage of total dose to the right and left hand for each procedure for the radiochemists involved in the production of ^68^ Ga-DOTA-TATE and ^99m^Tc.

In the case of ^99m^Tc, the highest percentage contribution to total *H*_p_(0.07) dose per unit activity was measured during the labelling procedure (84%) for the right hand of radiochemists. In contrast, during the production of ^68^ Ga-DOTA-TATE the highest percentage contribution to total *H*_p_(0.07) dose per unit activity was measured for the left hand of radiochemists during the dispensing procedure (61%). It is worth mentioning that in both cases the procedure which showed the lowest contribution to total dose (below 20%) was the elution procedure. The p-values obtained for the elution, labelling and dispensing procedures showed that the observed differences between ^68^ Ga-DOTA-TATE and ^99m^Tc were statistically significant for the labelling and dosing procedures.

Another popular radioisotope used in positron emission tomography is ^18^F. Similar to ^68^ Ga, the annihilation process of positrons generates gamma rays that are the main source of workers’ radiation exposure. However, the radiopharmaceutical ^18^F-FDG is produced automatically. Therefore, the only direct manual contact of workers’ hands with an open source of ^18^F activity is the quality control procedure of the produced ^18^F-FDG.

**Fig. 10:**
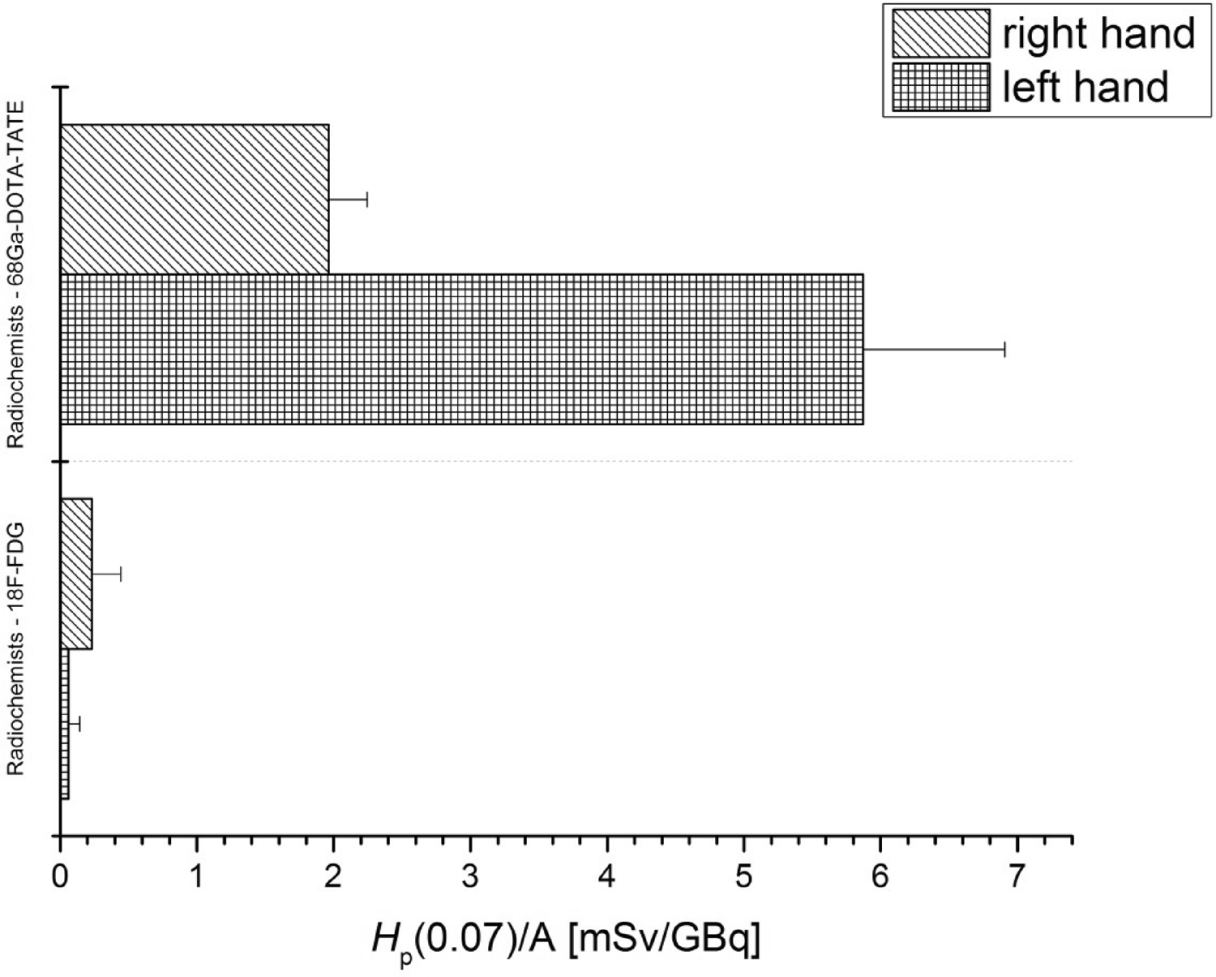
Average values of *H*_p_(0.07) doses per unit activity (A) (with standard errors) measured for radiochemists during the dispensing the ^68^ Ga-DOTA-TATE and ^18^F-FDG activity to patients

Hand exposures during the ^68^ Ga-DOTA-TATE and ^18^F-FDG dose dispensing procedures and ^18^F-FDG routines were also compared (Fig. 10).

As already said before, in the case of chemical compounds labelled with ^99m^Tc dosing is a manual process. Time required to complete the ^99m^Tc procedure is presented in Wrzesień (2018). In the case of ^18^F-FDG, the procedure of dose dispensing is fully automatic. The only direct involvement of a worker includes placing the syringe in the right place in the dispensing chamber before the syringe is automatically filled with the appropriate radiopharmaceutical activity, and removing the syringe from the dispenser after the dispensing process. Data presented in Fig. 10 show that during dose dispensing with ^18^F-FDG, the left hands of the investigated radiochemists were more exposed than the right ones, but the mean *H*_p_(0.07) dose per unit activity did not exceed 1 mSv/GBq. For radiochemists performing the same procedure with ^68^ Ga-DOTA-TATE, the situation is quite different. In this case the left hands of the involved radiochemists were exposed to a significantly higher dose. Specifically, the mean values of *H*_p_(0.07) per unit activity exceeded 5 mSv/GBq. In contrast, for the right hands, the mean *H*_p_(0.07) doses per unit dose did not exceed 2 mSv/GBq.

**Fig. 11:**
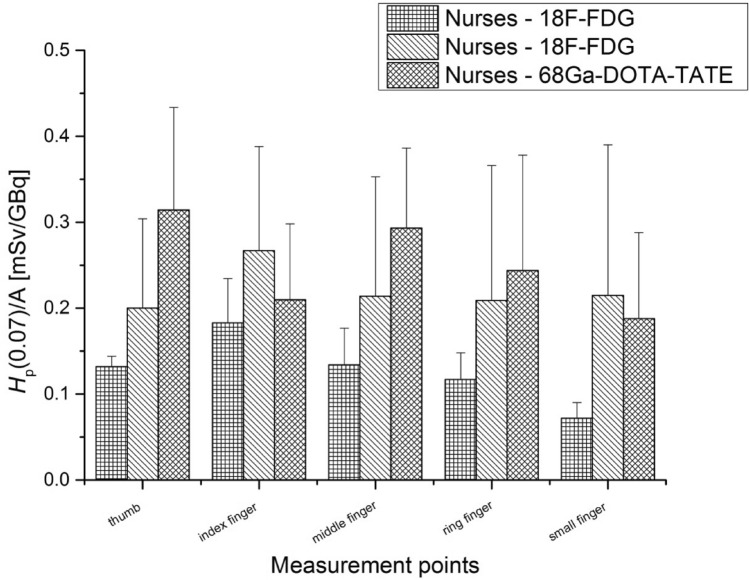
Average *H*_p_(0.07) doses per unit activity (A) (with standard errors) measured for the right hand of nurses during the injection of ^68^ Ga-DOTA-TATE and ^18^F-FDG

**Fig. 12:**
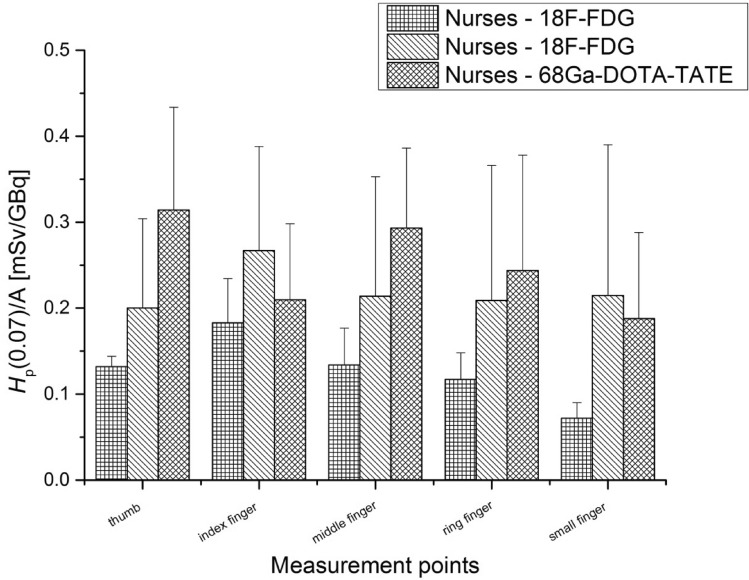
Average *H*_p_(0.07) doses per unit activity (A) (with standard errors) measured for the left hand of nurses during the injection of ^68^ Ga-DOTA-TATE and ^18^F-FDG

The final step in preparing the patient for PET examination is the intravenous injection of the radiopharmaceutical. In Figs. 11 and 12, exposures of the left and right hand fingertips of nurses who injected ^68^ Ga-DOTA-TATE are compared to those of nurses who injected ^18^F-FDG.

As was the case for the investigated radiochemists, the left hands were more exposed in the injection process for both ^68^ Ga-DOTA-TATE and ^18^F-FDG than the right hands. It is important to mention that all nurses who took part in the both studies were right-handed. For nurses during the injection of ^68^ Ga-DOTA-TATE, the average *H*_p_(0.07) doses per unit activity values were twice as high for the thumb and index finger as for the nurses during ^18^F-FDG injection. Similarly, the exposure resulting from working with a radiopharmaceutical based on ^68^ Ga was higher for the thumb and middle finger of the left hand.

## Assessment of the annual exposure of workers working with the radiopharmaceutical ^68^ Ga-DOTA-TATE

### Assessment of exposure of the hands of radiochemists

Assessment of exposure of fingertips was performed taking into account one-third of the number of working days per year (260) and the average dose of individual finger tips where the measurements had been performed, taking into account the average ^68^ Ga activity estimated for the individual procedures performed by the radiochemists handling ^68^ Ga-DOTA-TATE.

With this assumption, the maximum annual finger dose of 1,030 mSv was obtained for the index finger of the left hand during the dosing of the ^68^ Ga-DOTA-TATE activity. In contrast, the minimum estimated annual finger dose was 8 mSv for the middle finger of the right hand during generator elution. It is worth emphasizing, above all, that the maximum estimated dose is more than twice as high as the permissible annual dose limit, which is even more important given the fact that work with ^68^ Ga is only part of work of the investigated radiochemist, who also prepare other radiopharmaceuticals based on ^68^ Ga (such as PSMA) and additionally work with many other radionuclides, which consequently increases the annual doses to their hands further.

### Assessment of exposure of the hands of nurses

The assessment of the annual exposure of the hands of the investigated nurses was made based on the same assumptions as that for radiochemists. As a result, annual finger dose values in the range from 22 to 76 mSv (in both cases it concerns the index finger of the left hand) were obtained.

## Conclusions

The production procedure of the radiopharmaceutical DOTA-TATE labelled with ^68^ Ga includes a series of manual procedures, which include generator elution, labelling a chemical compound with ^68^ Ga, dosing of activity for the individual patients, and injecting the prepared activity of radiopharmaceutical into individual patients. The analysis of the results of dosimetric measurements performed for individual production stages leads to the following conclusions:

- In the case of the production of ^68^ Ga-DOTA-DATE, in the worst case a maximum *H*_p_(0.07) dose per unit activity of 49.36 ± 4.95 mSv/GBq was measured for one of the radiochemists during the dispensing procedure. In the case of nurses, a maximum *H*_p_(0.07) dose per unit activity of 1.28 ± 0.13 mSv/GBq was measured during the injection procedure;
- For both radiochemists and nurses, the greatest exposure to ionizing radiation was found for the non-dominant left hand. Consequently, the authors of the study recommend routine placement of a ring dosimeter on the middle finger of the non-dominant hand;
- It has been shown that the main element affecting recorded dose levels is the time spent working with the radioisotope. Therefore, the authors of the study recommend that personnel involved in the production of 68 Ga-DOTA-TATE should participate in periodic training, in order to reduce the time spent working with the open source radiation;
- The authors of the study also suggest paying attention to the level of complexity of manual operations performed in the production of other radiopharmaceuticals using short-lived radionuclide generators, which would lead to an increase in the exposure of workers' hands in particular;
- Comparison of the production procedures implemented for ^99m^Tc-labelled and ^68^Ga-DOTA-DATE radiopharmaceuticals showed that the step that dominated finger exposure was the labelling procedure in the case of ^99m^Tc-based radiopharmaceuticals, while in the case of ^68^ Ga-DOTA-DATE it was the dispensing procedure which accounted for approximately 60% of the total dose to fingertips radiochemists;
- Because production of ^68^ Ga-DOTA-DATE is more complex than that of ^99m^Tc-based radiopharmaceuticals, resulting doses to the fingertips are higher for procedures involving ^68^ Ga than for those involving ^99m^Tc;
- The maximum estimated exposure of the fingertips of the non-dominant hand was, in the worst case, twice as the annual permissible dose limit set at 500 mSv, for ^68^ Ga-DOTA-DATE procedures.

## Data Availability

Data supporting the results of this study are available upon request from the corresponding author [L. Albiniak]. The data are not available to the public due to the limitations of the agreements between the scientific institution and the medical institutions in which the dosimetry tests were carried out (among other things, this refers to the processing of data in a way that prevents the identification of the medical institution or the anonymization of the data of the employees participating in the measurements carried out).
